# Supplementary material for: Genome-Wide Association Study Identifies a Rice Panicle Blast Resistance Gene Pb3 Encoding NLR Protein
Source: Int J Mol Sci. 2022 Nov 14;23(22):14032. doi: 10.3390/ijms232214032 (PMC9698523; doi:10.3390/ijms232214032)
Supplement: Supplementary file 1 [file ijms-23-14032-s001.zip › ijms-2004179-supplementary.pdf]

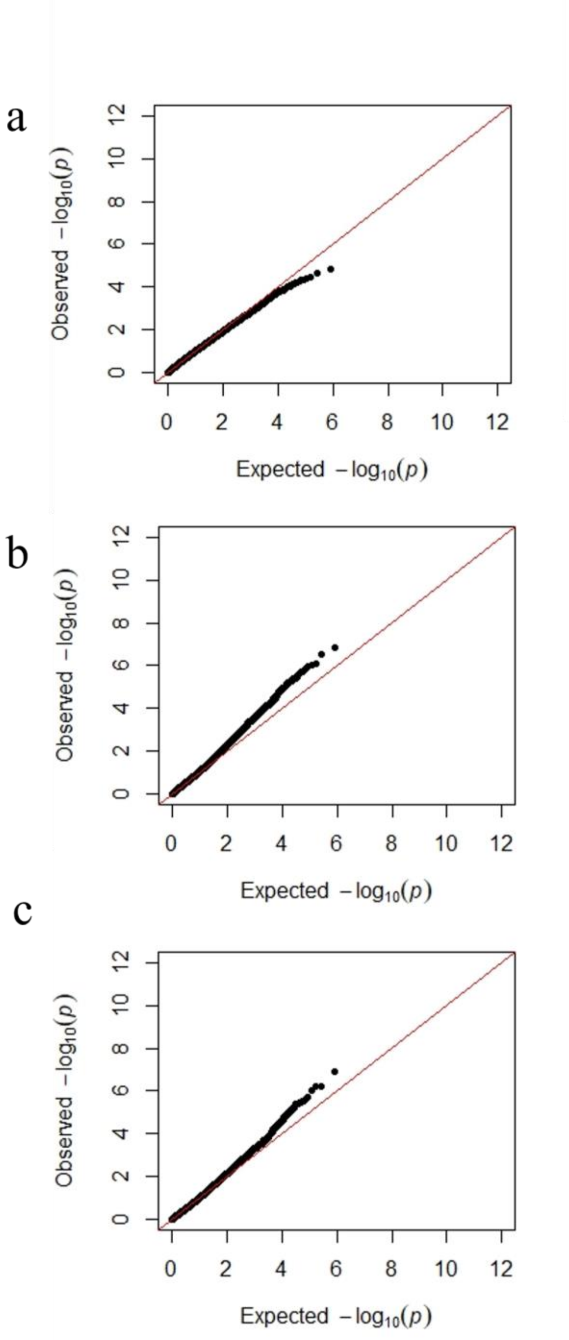

Figure S1 Quantile-quantile (Q-Q) plots for the GWAS analysis of panicle blast resistant loci. (Q-Q) plots show the fitness of selected models for different traits data (a)2017,(b)2018, (c)2019. X-axis: expected − Log 10 P; Y-axis: observed − Log 10 (P).

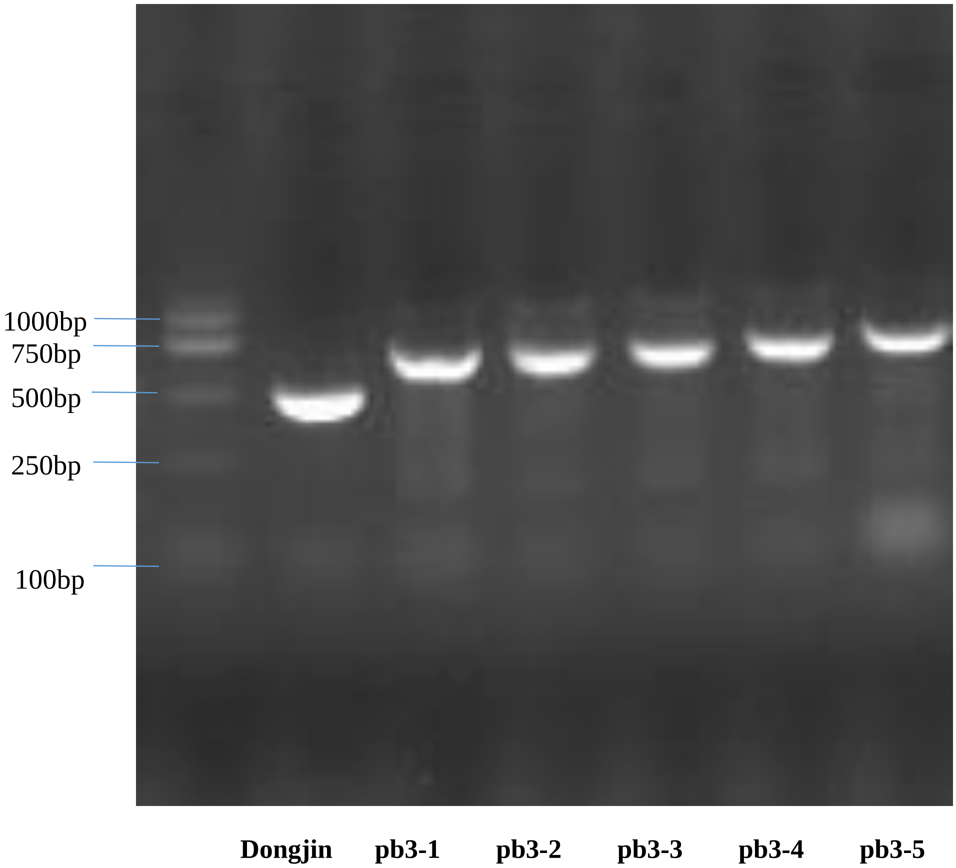

Figure S2 Positive verification of mutants. We designed primers at both ends of the T-DNA and the insertion position to verify the positive results of the mutant material.

Table S1. Primers used in this study.

Marker	Forward(5' TO 3')	Reverse(5' TO 3')	Annotation
44890q	CAACCTGTTGACTCGTGATGGC	ACGCGTAAATGCTCCTGGTGAAG	Primer for expression analysis of *LOC_Os11g44890*
44910q	AGAGCTTCAAGGTTGCAATGGAG	TTGCCTCCGGCATTCTGTCTTC	Primer for expression analysis of *LOC_Os11g44910*
44930q	CATGGGATTGAGGGCATTGAGC	ATTGGGCATCTCACCGTGTGTC	Primer for expression analysis of *LOC_Os11g44930*
44950q	CACTGCCACTGTCTCTACAAAGG	CCAATAGCAATGTGATGCCTTTCC	Primer for expression analysis of *LOC_Os11g44950*
44990q	GCTTGAAGCGTTGGTCATTGAGG	TTCAGGGTTCTGACACCACTCC	Primer for expression analysis of *LOC_Os11g44990*
45030q	TCTCCCTGCACATTGACCAAGC	GTACTTGCCCTTGAGCCACTTC	Primer for expression analysis of *LOC_Os11g45030*
Pb3q	GAGGTTGGATCTTTGGGACTGC	AGCTCCTTCAAGTTGGTCTGTCC	Primer for expression analysis of *Pb3*
CRISPR-testing	GGGTATTTCTTTGGTTGTAG	TCAACTTGTGTATCTTATAA	Primer for testing the editing type of CRISPR Pb3 gene
50885q(Actin)	AGGAAGGCTGGAAGAGGACC	CGGGAAATTGTGAGGGACAT	Internal control for expression analysis
Pb3-CRISPR-gRNA	GATGCCTTGTTTGAGGAGGT	TCAAGTTGGTCTGTCCAAGC	Primer for *Pb3* gene CRISPR vector
Pb3-T-DNA	ATCGATTTGGCTAGTTTTGA	GTCTCAAAACAGAGGACGGG	Primer for positive verification of T-DNA mutants
TDNA-LB1	CTAGAGTCGAGAATTCAGTACA		Primer for positive verification of T-DNA mutants
